# Smartphone Addiction Prevalence and Its Association on Academic Performance, Physical Health, and Mental Well-Being among University Students in Umm Al-Qura University (UQU), Saudi Arabia

**DOI:** 10.3390/ijerph19063710

**Published:** 2022-03-21

**Authors:** Mohammad Saud Alotaibi, Mim Fox, Robyn Coman, Zubair Ahmed Ratan, Hassan Hosseinzadeh

**Affiliations:** 1 Department of Social Work, College of Social Sciences, Umm Al-Qura University, Mecca 24382, Saudi Arabia; msmasoud@uqu.edu.sa; 2 School of Health and Society, Faculty of the Arts, Social Sciences and Humanities, University of Wollongong, Northfields Ave., Wollongong, NSW 2522, Australia; mfox@uow.edu.au (M.F.); rcoman@uow.edu.au (R.C.); zar241@uowmail.edu.au (Z.A.R.); 3 Department of Biomedical Engineering, Khulna University of Engineering and Technology, Khulna 9203, Bangladesh

**Keywords:** smartphone addiction, university students, academic performance, physical and mental well-being

## Abstract

Smartphone use can lead to smartphone addiction, which is a growing concern worldwide. However, there are limited studies about smartphone addiction and its impacts on university students in Saudi Arabia. This study aims to fill this gap. This is a quantitative study conducted among undergraduate students in Umm Al-Qura University (UQU), Saudi Arabia from May 2019 and February 2021. Study data were collected using both online and hard copy administered surveys. A self-administered questionnaire, Grade point average, Smartphone Addiction Short Version, and Kessler Psychological Distress scales were used to assess the outcomes. A total of 545 undergraduate students, mostly females, aged ≤21 years old and lived with large family sizes. More than half owned a smartphone for 5–8 years and the majority used their smartphone on average 6–11 h per day for social networking (82.6%), entertainment (66.2%) and web surfing (59.6%). Most of the participants were smartphone-addicted (67.0%). Logistic regression analysis showed that age ≤ 21, not gainfully employed, small family size and high family income were the main significant socio-demographic predictors of smartphone addiction. Smartphone-addicted participants were more likely to: have lower academic performance (GPA); be physically inactive; have poor sleep; be overweight/obese; have pain in their shoulder (39.2%), eyes (62.2%) and neck (67.7%) and have a serious mental illness (30.7%). This finding has significant implications for decision makers and suggests that smartphone education focusing on the physical and mental health consequences of smartphone addiction among university students can be beneficial.

## 1. Introduction

In recent years, smartphones have become an essential part of daily lives globally [1]. The mobile industry has improved significantly in terms of technological advancement since the release of the first publicly available smartphone in 2007 [2]. Technological advancements have evolved smartphones and made them inexpensive and user-friendly, paving the way for large-scale use. This has led to an exponential increase in smartphone use worldwide. In 2018, there were more than 2.5 billion smartphone users globally [3] and this figure is expected to reach 7.516 billion by 2026 [4]. Smartphone may improve productivity (e.g., email), promotes social interaction (e.g., social media), expands entertainment options and grants access to information and services (e.g., online banking) [2]. Despite the benefits, smartphones can potentially lead to “excessive” or “compulsive” use, which has been referred to as “smartphone addiction” [2].

Smartphone addiction is a worldwide growing concern [5]. Different terms have been used to described smartphone addiction such as “smartphone overuse”, “mobile phone addiction”, “problematic mobile phone use” [6], “addiction proneness” [7] and “excessive use of smartphones” [8]. Recent literature on smartphone addiction has categorized the smartphone addiction as a behavior addiction [9].

Alavi et al. [10] reported “the concept of addiction is not easy to define, and the usage of the term addiction has been considered controversial”; however, central to its definition is the dependence on a substance or activity, according to Ching et al. [11], smartphone addiction is “mainly characterized by excessive or poorly controlled preoccupations, urges, or behaviors regarding smartphone use, to the extent that individuals neglect other areas of life” (p. 2).

Smartphone use is increasing exponentially among university students worldwide [12]. Millennials (aged 18 to 34) are much more likely to be smartphone users [13]. A study conducted a global mobile consumer survey among more than 51,000 participants from 32 countries showed that the 93% of participants aged 18 to 24 years old had the highest smartphone ownership and spent more time on a smartphone [14]. Other studies conducted among university students in various countries found that smartphone ownership among students was very high [15,16,17,18,19,20,21]. These young adults use their devices for browsing the internet, watching videos or checking updates in social networking sites (SNSs) [22]. The smartphone addiction could be represented as nomophobia [23], phubbing [24,25] or social media addiction [26]. A recent systematic review revealed that smartphone addiction could be detrimental to physical and mental health [27], interestingly, another review reported that social media addiction found a negative but small relationship between social media addiction measures and well-being [28].

Smartphones have provided them the opportunity to learn useful things anytime and anywhere. Teachers also use these devices for arranging discussion sessions and retrieving information about the performance of the students [29]. However, it has also been observed that instead of paying attention to the teacher’s lectures, college students remain busy with texting, and are unable to recall what the teachers said [30]. Furthermore, smartphone addiction blocks face-to-face communication, which is vital for university students while communicating with their teachers and peers [31]. It also reduces their level of concentration during a typical class, and even leads to various physical and mental problems [32].

## 2. Factors of Smartphone Addiction

Most studies conducted among university students found that younger age students are at higher risk of smartphone addiction [33,34,35,36]. For instance, a study conducted among 198 college students in Austria found that younger students had higher levels of smartphone addiction [34]. This is because younger ages are more likely to adopt new technologies than older groups [37] making them more vulnerable to smartphone addiction.

Previous studies also found that family monthly income was associated with smartphone addiction among university students [38,39,40,41]. A study among 1062 Iranian undergraduate students revealed there was an association between high family income and smartphone addiction. However, another study conducted by Akturk and Budak [41] among 1149 Turkish university students, showed that lower income was associated with smartphone addiction. Employment status was also associated with smartphone addiction. Lopez-Fernandez [42] found that unemployed group were more likely to be smartphone-addicted.

Smartphone-related interactive applications can increase the risk of smartphone addiction among university students. The digital revolution allows this kind of technology to be available for everyone and to be more attractive. For instance, the results of a study among 2367 university students showed that almost 95% of participants used a smartphone to check social media applications [43]. Another example from the USA showed that social media applications significantly increased the risk of smartphone addiction [44]. Recently, it was suggested that social media and game addiction may be the subsets of smartphone addiction [45]. According to Muhammad, Schneider [46], smartphones often encourage people to be involved with constant use of social media, excessive texting, video gaming and online shopping. This study concluded that social media and gaming applications were more likely to lead to excessive smartphone use.

## 3. Impacts of Smartphone Addiction

Studies have shown that deterioration in academic performance was one of the most frequently reported adverse outcomes of smartphone addiction among university students in the United States [47] and Italy [48]. In addition, other studies showed the effect of overusing smartphones on productivity can be related to daily interruptions [49,50] which include work-related and non-work-related productivity [50].

Studies have revealed that less physical activity, body pain and lack of sleep were related to smartphone addiction. Studies from the USA [51] and Korea [52], reported that excessive smartphone use had adverse effects on students’ physical health. Several studies showed that overusing smartphones could be related to having body pain among university students [53]. Another study found that university students’ sleeping quality was negatively impacted by smartphone overusing [43,54,55].

In addition, numerous studies found that smartphone addiction was associated with stress, anxiety, and negative emotions [56,57]. A cross-sectional study conducted by Samaha and Hawi [56] in Lebanon among 249 university students showed that smartphone addiction risk was positively associated to perceived stress. Of the participants, 53.4% who were at risk of smartphone addiction were having high levels of stress. Recently, the results from a study among 525 Malaysian university students from three public universities, showed a significant positive correlation between smartphone addiction with depression, anxiety and stress [57].

## 4. Aims

In Saudi Arabia, the rate of smartphone usage increased from 61.54% in 2015 to 65.18% in 2018 and is projected to reach 66.28% in the year 2022 [58]. Smartphone usage in particular among the country’s younger population has increased substantially due to access to the social media [59]. For instance, over half (54%) of Saudis were active social media users with smartphones [60]. The average use of smartphones in Saudi Arabia ranged from two to six hours per day in 2016 [61]. There are about 36 universities with over 1.7 million enrolled students in Saudi Arabia [62], however there are limited studies about smartphone addiction among university students in the country.

This study aims to fill this gap by (1) exploring the prevalence of smartphone addiction, (2) analysing the association of sociodemographics, smartphone ownership, smartphone daily use and purpose of smartphone use and smartphone addiction among the participants, (3) and examine the differences between smartphone addiction group and non-addiction on academic performance, physical health, and mental well-being among university students in Umm Al-Qura University (UQU), Saudi Arabia.

In particular, we hypothesized that:

**Hypothesis** **1** **(H1).** *The prevalence of smartphone addiction is high among university students.*

**Hypothesis** **2** **(H2).** *Sociodemographic variables such as gender, age, gainfully employment status, marital status, family income and family size can predict smartphone addiction.*

**Hypothesis** **3** **(H3).** *Smartphone usage variables (average hours of daily using smartphones and purpose of smartphone usage) can predict smartphone addiction.*

**Hypothesis** **4** **(H4).** *University students who are addicted to smartphone are more likely to gain lower Grade Point Average.*

**Hypothesis** **5** **(H5).** *University students who are addicted to smartphone are more likely to have poor physical health status such as physical inactivity, less sleep hours, experience pain or discomfort in body parts while using a smartphone, and being overweight/obese.*

**Hypothesis** **6** **(H6).** *University students who are addicted to smartphone are more likely of having a serious mental illness.*

## 5. Methods

### 5.1. Study Design and Data Collection

This was a cross-sectional study conducted among undergraduate students from UQU, Saudi Arabia from June 2019 and February 2021. A power analysis using Cohen’s formula [63] indicated that a sample size of 304 would give a 95% chance of detecting correlation ± 0.10 at 0.05 level, allowing for 15% incomplete surveys. The effect size of ±0.10 was selected as the smallest effect to detect. However, a total of 545 students were participated in this study. Data were collected using a self-administered questionnaire. The survey was available both online and in hard copies (paper) to improve data collection and recruit a large number of students in this study. An online version of the survey was administered using Google Forms. The study was advertised through university social media platforms, flyers, and student emails. The online survey link was added to the study advertisement. Email reminders were sent out at intervals of two weeks by UQU. The online survey included the Participant Information Sheet (PIS) and Participant Consent Form (PCF). Students were also invited to complete the survey in a hard copy format. A list of eligible students from different courses was extracted from course enrolment data. Students were provided with a hard copy survey, PIS and PCF and were invited to complete the hard copy version of the survey and return it to the boxes provided in teaching buildings and outside education offices in different schools/faculties. The survey took approximately 20 min to complete.

### 5.2. Study Measures

#### 5.2.1. Sociodemographic

Sociodemographic data included gender; age; gainfully employment status; family income; marital status; family size; and semesters of study.

#### 5.2.2. Smartphones Use Data

Smartphones use data included duration of smartphone ownership (years), average hours of using smartphone daily, and purpose of daily smartphone use. Regarding purpose of smartphone use, participants were asked to report their frequency of using smartphone in their daily usage (i.e., social networking, entertainment, web surfing, education, games, shopping…) from 1 (never) to 5 (always) on a Likert scale.

#### 5.2.3. Academic and Physical Health Information

Academic performance measured by asking the participants to report their overall Grade Point Average, then it is categorized into four level (excellent, very good, good, and pass) according to GPA and grades regulation at Umm Al-Qura University [64]. Physical health information included physical activity status; sleeping hours which categorized into recommended and not recommended times according to National Sleep Foundation [65]; pain or discomfort while using a smartphone; and Body Mass Index (BMI) was calculated by using participation’s weight and height data.

#### 5.2.4. Smartphone Addiction

Smartphone addiction was assessed using the Smartphone Addiction Scale (SAS-SV), which is an internationaly validated smartphone addiction scale [66]. The SAS-SV (Cronbach’s alpha = 0.911) includes 10 questions each scoring from 1 (strongly disagree) to 6 (strongly agree) on a Likert scale. An overall SAS-SV score ranges from 10 to 60, a higher score indicating more problematic smartphone use. The cut-off value of being smartphone-addicted differes between genders. For males, the cut-off value of being smartphone-addicted is 31 while it is 33 for females. An Arabic language version of the SAS-SV with a Cronbach’s alpha vale of 0.91) was used in this study [67].

#### 5.2.5. Mental Well-Being

Mental well-being was assessed using Kessler Psychological Distress Scale (K-6) developed by Kessler et al. [68,69]. This scale is used to assess non-specific psychological distress by predominantly examining anxiety and depression symptoms [70]. It consists of six items about personal feelings in past 30 days including “nervousness”, “hopeless”, “restless or fidgety”, “so sad that nothing could cheer you up”, “everything was an effort” and “worthless”. Each item scores from zero to four (zero = none of the time, one = a little of the time, two = sometimes, three = most of the time, and four = all of the time). This scale score ranges from 0 to 24, the cut-off score of a serious mental illness is ≥13. An Arabic language (Cronbach’s alpha = 0.81) [71], which was used in this study.

### 5.3. Data Analysis

Data were analyzed using IBM SPSS Statistics Version 27.0. Univariate statistics were generated to determine the prevalence of smartphone addiction and describe sociodemographic, smartphones use data, academic performance, physical health and mental well-being. Regression analyses were performed to determine whether the independent variables (sociodemographic and smartphones use data (“smartphone ownership, smartphone daily use, purpose of smartphone use”) can predict smartphone addiction. To find main predictors of smartphone addiction, forward stepwise logistic regression analyses were performed. The forward stepwise regression model keeps adding the most significant variables into the model until none of the remaining variables can improve the model [72]. Chi-square analyses were used for analyzing the effect of smartphone addiction on academic performance, physical health, and mental well-being. Cramer’s V and phi coefficients were used to estimate effect sizes when the chi-square test resulted as statistically significant.

## 6. Results

### 6.1. Demographic

A total of 545 undergraduate students from Umm Al-Qura University participated in this study. As outlined in Table 1, over half of the participants were females (54.5%), 39.8% were aged ≤21 years old and 41.3% were from a large family size (eight members or more). The majority of the participants were not employed gainfully (85.3%), single (86.2%) and from a low family income (58.7%). Most of the participants were in semesters 5–8 of their study (56.3%).

### 6.2. Smartphone Ownership, Daily Use, and Purpose of Use

As shown in Table 2, more than half of the participants (50.8%) owned a smartphone for 5–8 years. Majority of the participants used their smartphone on average 6–11 h per day.

In terms of the purpose of daily smartphone use, as outlined in Table 3, most of the participants reported they were frequently and always using their smartphone for social networking (82.6%), entertainment (66.2%) and web surfing (59.6%).

### 6.3. Smartphone Addiction Prevalence

The prevalence of smartphone addiction among participants was 67.0% (59.3% in male and 73.4% in female participants).

### 6.4. Academic, Physical Health and Mental Well-Being

More than one-third of the participants academically performed “good” or did “pass” (see Table 4). Over half of the participants were sometimes physically active (60.4%) and close to one-third were physically inactive (29.2%). Over 30% half of the participants slept less than 6 h per day, which is less than the recommended 7 to 9 h per day. Over one-third of the participants were overweight or obese. The participants experienced pain in their neck (61.3%), eyes (57.8%), hand (49.5%) and shoulders (36.1%). Over a quarter of participants recorded having a probable serious mental illness (26.1%).

### 6.5. Associations of Sociodemographic with Smartphone Addiction

As outlined in Table 5, forward stepwise logistic regression analysis model was statistically significant (χ2 (4) = 52.36; *p* < 0.001) and explains approximately 12.7% of the variance according to Nagelkerke’s R2. The model showed age ≤ 21, not gainfully employed, small family size and high family income were the main significant socio-demographic predictors of smartphone addiction. Participants aged ≤21 were 2.64 times more likely to be smartphone-addicted compared to their counterpart aged ≥24 (OR = 2.64, 95% CI: 1.75–4.00). Similarly, gainfully unemployed participants were 2.22 times more likely to be smartphone-addicted compared to those who had a part or full-time gainful job (OR = 2.22, 95% CI: 1.35–3.66). Participants from a small family size ≤ 4 were 1.76 times more likely to be smartphone-addicted compared to those living with a large family ≥8 (OR = 1.76, 95% CI: 1.08–2.87). Participants with a high family income SAR ≥ 15,000 were 1.74 times more likely to be smartphone-addicted than those with a low family income SAR < 10,000 (OR = 1.74, 95% CI: 1.03–2.92).

### 6.6. Associations of Smartphone Use Data with Smartphone Addiction

Forward stepwise logistic regression analysis model was statistically significant (χ2 (4) = 113.33; *p* < 0.001) and explains approximately 26.1% of the variance according to Nagelkerke’s R2 (see Table 6). The model found that average daily use of more than 6 h, entertainment, and social networking were the main predictors of smartphone addiction among the participants. Participants using a smartphone on average 6–10 h per day were 2.26 times more likely to be smartphone-addicted (OR = 2.26, 95% CI: 1.47–3.47) whereas those who used on average ≥ 11 h per day were 6.98 times more likely to be smartphone-addicted (OR = 6.98, 95% CI: 3.26–13.48). Participants using a smartphone for social networking and entertainment were 1.71 times (OR = 1.71, 95% CI: 1.37–2.13) and 1.43 times (OR = 1.43, 95% CI: 1.16–1.76) more likely to be smartphone-addicted, respectively.

### 6.7. Comparison of Academic, Physical Health and Mental Well-Being between Smartphone-Addicted and Non-Addicted Groups

Table 7 shows that participants within smartphone-addicted group were less likely to have excellent, very good or good GPA, an indicative of academic performance, compared to those within non-smartphone-addicted group (*X*^2^ = 14.97, *p* = 0.002). For instance, the rate of pass GPA within smartphone-addicted group was 14% while it was 3.3% within non-smartphone-addicted group. Participants within smartphone-addicted group were more likely to be physically inactive (34.0%), sleep less than 6 h per day (36.4%) and be overweight/obese (41.1%). Smartphone-addicted participants were also more likely to have pain in their shoulder (39.2%), eyes (62.2%), and neck (67.7%) compared to non-addicted participants. In terms of mental well-being, the chance of having a serious mental illness within smartphone-addicted group was much greater (30.7%) compared to non-addicted group (16.7%).

## 7. Discussion

### 7.1. Smartphone Addiction Prevalence

As we expected of our hypothesis (H1), the study results revealed that 67% of the participants were smartphone-addicted using SAS-SV (Smartphone Addiction Scale-Short Version) [66]. This is in line with the findings of a cross-sectional study among dental students in Saudi Arabia, which found that that 71% of the students were smartphone-addicted [73]. However, studies using the same measurement (SAS-SA) in other countries reported lower rates of smartphone addiction among young people in China (29.8%) [1], Brazil (33.1%) [74], Turkey (39.8%) [55], Lebanon (44.6%) [75] and Malaysia (46.9%) [11]. The high prevalence of smartphone addiction among the participants in this study might be explained by the findings of a study reporting that young people including students in Saudi Arabia are increasingly using smartphones for exchanging news and knowing what is happening in the country and their community [21]. Another possible explanation of the high prevalence of smartphone addiction among the participants might be related to the growing rate of smartphone use among young people including students due to access to the internet, which is widely available in Saudi Arabia, to watch movies, listen to music, and access to different social media platforms [59]. Further, in the Saudi Arabia smartphone is considered a sign of keeping up with global modernization [21].

### 7.2. Sociodemographic and Smartphone Addiction

In line with the literature and hypothesis (H2), young adults (≤21 years) [34], those who were not gainfully employed [76], those living with a small family [77] and those from a high-income family [38,39,40,78] were more likely to be smartphone-addicted, however, gender and marital status from hypothesis (H2) were not related to smartphone relation. Our results suggest that young adults are more likely to embrace and overuse a smartphone [37]. Small family size has been associated with smartphone addiction due to a high rate of using social network sites [77] and internet use [79]. Further, small families might not offer equivalent opportunities for socialization as extended families therefore the members are more likely to use the internet through their smartphone to explore further socialization opportunities [80]. The association of smartphone addiction with not being gainfully employed among university students suggests that this cohort may have more free time [81], which may lead to a smartphone overuse [76]. The correlation of high family income with smartphone addiction in this study recommends that the financial affordability of family may contribute to smartphone overuse or addiction among university students. This finding suggests that affordability and easy access to mobile phones may lead to smartphone addiction [38]. Similar findings have been reflected in other studies [38,39,40].

In terms of gender, there was not any association with gender and smartphone addiction in this study. Some studies found that female students were more likely to be smartphone-addicted compared to male students [66,78,82]. However, other studies found male students were at high risk of smartphone addiction [33,83]. Meanwhile, some studies did not find any gender difference in smartphone addiction [1,84,85]. These inconsistent findings warrant further studies whether gender is a significant factor in smartphone addiction.

Our findings suggest that being young (≤21) and not gainfully employed along with high family income and small family size (≤4) are significant risk factors or indicators of high chance of smartphone addiction among university students. These findings require further studies and most of all, define better who are the students more vulnerable to smartphone addiction. Further, these cohorts should be prioritized in any smartphone addiction prevention campaigns.

### 7.3. Smartphone Use Data and Smartphone Addiction

As we expected from (H3), smartphone-addicted participants were more likely to use smartphone for more than 6 h as well as using a smartphone for entertainment and social networking. Similarly, literature suggests that spending more time on a smartphone [86] with a high frequency [87] is more likely to increase the chance of smartphone addiction [88]. Previous studies also found that using smartphone for social networking, entertainment and gaming is more likely to lead to smartphone addiction [89]. Understandably, higher levels of social media engagement require using smartphones more frequently for a long time, which is more likely to increase the chance of smartphone addiction [90]. This finding has significant implications for health policy and decision makers in Saudi Arabia, where there are limited entertainment opportunities and social activities and university students are more likely to be encouraged to use smartphones to access further entertainment and social activities offered by the internet [91,92]. This also might explain the high rate of smartphone use for social networking (82.6%) and entertainment (66.2%) among the participants in this study.

### 7.4. Academic Performance, Physical Health, Mental Well-Being and Smartphone Addiction

Our results supported the hypothesis (H4) that smartphone-addicted participants were less likely to have excellent, very good or good academic performance compared to non-smartphone-addicted participants. Consistent with literature, this finding indicates that smartphone addiction can lead to lower academic achievement among university students [75,93]. One explanation could be that smartphone addiction may distract students’ attention away from academic tasks [94]. As we expected from our hypothesis (H4), participants within smartphone-addicted group were more likely to be physically inactive and overweight or obese compared to those within non-addicted group. Consistently, Kim et al. [52] conducting a study among university students in China found that smartphone-addicted participants were less physically active. In relation to being overweight or obese, in line with our findings, a recent study found that smartphone addiction was correlated with body mass index and eating disorders among college students [95]. Further a cross-sectional study in Saudi Arabia found that smartphone usage was associated with eating more fast food and gaining weight among university students [43].

In terms of sleeping pattern, in line with previous studies [55,96,97] and confirming our hypothesis (H5), the results from this research showed that smartphone-addicted participants were more likely to sleep less than 6 h per day compared to none addicted ones. This might be because smartphone addiction is more likely to result in bedtime procrastination. Bedtime procrastination is a relatively new concept, which is a possible cause for insufficient sleep. The smartphone addicts may find it difficult to stop using phones before going to bed and this may initiate higher levels of bedtime procrastination leading to shorter sleep duration and poorer sleep quality [98]. Further, the blue light emitted by a smartphone may have a negative effect on an individual’s circadian rhythms, leading to negative sleep consequences, such as going to bed later than intended thus reducing overall sleep time [99].

As we expected from our hypothesis (H4), smartphone-addicted participants were also more likely to experience pain in their shoulder (39.2%), eyes (62.2%) and neck (67.7%). Similarly, literature showed that neck and hand pains [100] and visual fatigue [101] were associated with smartphone addiction. Further, smartphone addiction induced neck and shoulder pains may result in musculoskeletal disorders in a long run [102]. Continuous use of a smartphone can also cause defective postures causing pain in different parts of the body [103]. Other studies found that De Quervain tenosynovitis, pain on the wrist, is closely associated with different electronic devices [104]. Texting and chatting on a smartphone have been considered a risk factor for De Quervain tenosynovitis [105]. This finding suggests that smartphone education programs should discuss the physical consequences of smartphone addiction and overuse.

The results supported our hypothesis (H4) that smartphone-addicted participants were more likely to have serious mental illness (30.7%) compared to the non-addicted ones (16.7%). This is in line with the findings of review papers suggesting that stress, anxiety and depression are frequently associated with smartphone addiction [2,9]. Literature suggests that overusing social media, instant messaging, e-mail communication instead of in-person interactions is more likely to lead to social isolation [106] triggering stress, anxiety and depression among young adults [107,108]. Fear of Missing Out (FoMO) could also be an underlying cause of depression and anxiety related to smartphone addiction [109]. Addiction or overuse of a smartphone involves a tendency to check notifications at all times and such behavior patterns can lead to a “reassurance seeking” pathway [2,110,111], that ultimately results in FOMO.

## 8. Implications

The results of this study have significant implications for decision-makers in terms of the health and academic performance of university students, and highlights factors driving smartphone overuse and addiction among university students. Such findings can assist universities and government organizations to design effective smartphone addiction prevention programs in university settings. As such, the identified factors driving or influencing smartphone addiction overuse and addiction are of significant value for decision-makers in universities.

- Establish recreational services which encourage university students to engage in other leisure activities than their smartphone;
- Develop and implement various educational programs which raise awareness about smartphone addiction among university students;
- Develop policies and guidelines limiting the usage of smartphones during lectures;
- Establish free and accessible sports facilities in all universities.

## 9. Recommendations for Further Research

This study suggests that understanding of smartphone addiction among university students requires further studies as follows:

- Longitudinal studies to explain and confirm the causal relationship between the main factors predicted smartphone addiction in this study;
- Extending this undergraduate research to include postgraduates would advance an understanding of smartphone addiction across more comprehensive university settings in Saudi Arabia;
- Further studies are also required to interrogate why certain groups of university cohorts, for example female students, are more vulnerable to smartphone addiction.

## 10. Limitations

Despite invaluable data, this study has some limitations. The study employed a cross-sectional design hence identified significant relationships between tested independent variables and the dependent variable (smartphone addiction) cannot be inferred as causal. In addition, data on many tested independent variables were self-reported, which may subject to recall bias. Study data were collected using convenience sampling methods as such this study findings cannot be generalized to larger or similar populations.

## 11. Conclusions

This study’s findings suggest that smartphone addiction was prevalent among university students. Additionally, the finding showed that socio-demographic variables (age 21 or less, not gainfully employed, small family size and high family income) and average daily use of more than 6 h, entertainment and social networking were significant predictors of smartphone addiction. Furthermore, the results showed that smartphone addiction students more likely to had a lower GPA and poor physical health as well as having a serious mental illness compered to non-addicted students. This finding suggests that smartphone education focusing on physical and mental health consequences of smartphone addiction among university students can be beneficial.

## Figures and Tables

**Table 1:** Distribution of sociodemographic characteristics, N = 545.

	*n*	(%)
Gender		
Male	248	(45.5)
Female	297	(54.5)
Age ^a^		
≤21	217	(39.8)
22–23	198	(36.3)
≥24	130	(23.9)
Gainfully Employment Status ^b^		
Full/Part-time employed	80	(14.7)
Unemployed	465	(85.3)
Family Monthly Income ^c^		
Low income (<10,000 SAR)	320	(58.7)
Average income (10,000–15,000 SAR)	118	(21.7)
High income (>15,000 SAR)	107	(19.6)
Marital Status ^b^		
Single	470	(86.2)
Married	64	(11.7)
Others including divorced and widowed	11	(2.0)
Family Size ^a^		
Small (≤4)	122	(22.2)
Average (5–7)	198	(36.5)
Large (≥8)	225	(41.3)
Semesters of Study ^a^		
≤4	142	(26.1)
5–8	307	(56.3)
≥9	96	(17.6)

^a^ Age, family size and semesters of the study were continuous variables but they are categorized into groups for the purpose of this study. ^b^ Gainfully employment and marital status categories are recategorized due to small numbers in some original categories. ^c^ Family monthly income are recategorized into new groups namely low family income (SAR < 3000, 3000–7000, 7000–10,000); average family income (SAR 10,000–15,000) and high family income (SAR < 15,000) in line with Saudi family income data (https://bit.ly/34SVij2) (accessed on 20 January 2022).

**Table 2:** Distribution of smartphone ownership and daily use, N = 545.

	*n*	(%)
Years of Smartphone Ownership ^a^		
≤4	73	(13.4)
5–8	277	(50.8)
≥9	195	(35.8)
Average of Hours Using Smartphone Daily ^a^		
Average use (≤5)	192	(35.2)
More than average (6–10)	234	(42.9)
Higher than average (≥11)	119	(21.8)

^a^ years of smartphone ownership and average of hours using smartphone daily were continuous variables, but they are categorized into groups for the purpose of this study.

**Table 3:** Distribution of the purpose of daily smartphone use, N = 545.

	Never and Rarely		Occasionally		Frequently and Always	
	*n*	(%)	*n*	(%)	*n*	(%)
Social networking	29	(5.3)	66	(12.1)	450	(82.6)
Entertainment	50	(9.2)	134	(24.6)	361	(66.2)
Web surfing	81	(14.9)	139	(25.5)	325	(59.6)
Eduction	122	(22.4)	182	(33.4)	241	(44.2)
Games	193	(35.4)	129	(23.7)	223	(40.9)
Shopping	177	(32.5)	150	(27.5)	218	(40.0)
Map, navigation	169	(31.0)	180	(33.0)	196	(36.0)
Phone calls/text messages	161	(29.5)	202	(37.1)	182	(33.4)
Health	173	(31.7)	198	(36.3)	174	(31.9)
Religion	164	(30.1)	240	(44.0)	141	(25.9)

**Table 4:** Distribution of academic, physical health and mental well-being, N = 545.

	*n*	(%)
Overall Grade Point Average		
Excellent (3.50–4.00)	132	(24.2)
Very Good (2.75–3.49)	194	(35.6)
Good (1.75–2.74)	162	(29.7)
Pass (≤1.74)	57	(10.5)
Physical Activity		
I do not currently exercise	159	(29.2)
I exercise sometimes	329	(60.4)
I exercise regularly	57	(10.5)
Average of Sleep Hours		
≤6 h (Not Recommended)	178	(32.7)
7–9 h (Recommended)	288	(52.8)
≥10 (Not Recommended)	79	(14.5)
Body Mass Index (BMI)		
Underweight (≤18.4)	80	(14.7)
Healthy weight (18.5–24.9)	260	(47.7)
Overweight (25.0–29.9)	129	(23.7)
Obese (≥30.0)	76	(13.9)
Experienced Pain		
Shoulder		
Yes	197	(36.1)
No	348	(63.9)
Eyes		
Yes	315	(57.8)
No	230	(42.2)
Neck		
Yes	334	(61.3)
No	211	(38.7)
Hands		
Yes	270	(49.5)
No	275	(50.5)
Mental Well-Being (Kessler-6)		
Probable serious mental illness	142	(26.1)
No Probable serious mental illness	403	(74.9)

**Table 5:** Forward stepwise logistic regression analysis of associations of the sociodemographic variables with smartphone addiction, N = 545.

	OR	95% CI		*p*
		Lower	Upper	
Age				
<21 vs. ≥24	2.64	1.75	4.00	0.001
Gainfully Employment Status				
Unemployed vs. full time/part time employed	2.22	1.35	3.66	0.002
Family Monthly Income				
High income (SAR 15,000) vs. low income < (SAR 10,000)	1.74	1.03	2.92	0.037
Family Size				
Small ≤ 4 vs. large ≥ 8	1.76	1.08	2.87	0.022

Hosmer–Lemeshow Goodness-of-fit = 0.49, Model chi-square = 52.36, df = 4, *p* ≤ 0.001, Nagelkerke R Square = 0.127. OR = odds ratio, CI = confidence interval, A *p*-value ≤ 0.05 was considered significant.

**Table 6:** Forward stepwise logistic regression analysis of associations of smartphone ownership, daily use, and purpose of use with smartphone addiction, N = 545.

	OR	95% CI		*p*
		Lower	Upper	
Average of Hours Using Smartphone Daily				
More than avarage (6–10) vs. average use ≤ 5 h	2.26	1.47	3.47	0.001
More higher than average ≥ 11 vs. average use ≤ 5 h	6.98	3.62	13.48	0.001
Purpose of Use				
Entertainment	1.43	1.16	1.76	0.001
Social networking	1.71	1.37	2.13	0.001

Hosmer–Lemeshow Goodness-of-fit = 0.52, Model chi-square = 113.33, df = 4, *p* ≤ 0.001, Nagelkerke R Square = 0.261. OR = odds ratio, CI = confidence interval, A *p*-value ≤ 0.05 was considered significant.

**Table 7:** Comparison of academic, physical health and mental well-being information between smartphone-addicted and non-smartphone-addicted groups, N = 545.

	Non-Smartphone-Addicted		Smartphone-Addicted		df	*X*^2^(545)	*p*	Effect Size
	*n*	(%)	*n*	(%)				
Overall Grade Point Average					3	14.97	0.002	0.166
Excellent (3.50–4.00)	47	(26.1)	85	(23.3)				
Very Good (2.75–3.49)	72	(40.0)	122	(33.4)				
Good (1.75–2.74)	55	(30.6)	107	(29.3)				
Pass (≤1.74)	6	(3.3)	51	(14.0)				
Exercise Activity					2	12.93	0.002	0.154
I do not currently exercise	35	(19.4)	124	(34.0)				
I exercise sometimes	121	(67.2)	208	(57.0)				
I exercise regularly	24	(13.3)	33	(9.0)				
Average of Sleep Hours					2	7.17	0.028	0.115
≤6	45	(25.0)	133	(36.4)				
7–9	106	(58.9)	182	(49.9)				
≥10	29	(16.1)	50	(13.7)				
BMI					2	6.13	0.046	0.106
Underweight (≤18.4)	27	(15.0)	53	(14.5)				
Healthy weight (18.5–24.9)	98	(54.4)	162	(44.4)				
Overweight/Obese (≥25.0)	55	(30.6)	150	(41.1)				
Experienced Pain								
Shoulder					1	4.00	0.036	0.090
Yes	54	(30.0)	143	(39.2)				
No	126	(70.0)	222	(60.8)				
Eyes					1	8.74	0.003	0.127
Yes	88	(48.9)	227	(62.2)				
No	92	(51.1)	138	(37.8)				
Neck					1	19.00	0.001	0.187
Yes	87	(48.3)	247	(67.7)				
No	93	(51.7)	228	(32.3)				
Hands					1	1.26	0.261	--
Yes	83	(46.1)	187	(51.2)				
No	97	(53.9)	178	(48.8)				
Mental Illness (Kessler-6)					1	12.29	0.001	0.150
Probable serious mental illness	30	(16.7)	112	(30.7)				
No Probable serious mental illness	150	(83.3)	253	(69.3)				

A *p*-value ≤ 0.05 was considered significant.

## Data Availability

The data presented in this study are available on request from the corresponding author. The data are not publicly available due to privacy restrictions.
